# Predictive factors of para-aortic lymph nodes metastasis in cervical cancer patients: a retrospective analysis based on 723 para-aortic lymphadenectomy cases

**DOI:** 10.18632/oncotarget.16025

**Published:** 2017-03-08

**Authors:** Xiaotian Han, Hao Wen, Xingzhu Ju, Xiaojun Chen, Guihao Ke, Yuqi Zhou, Jin Li, Lingfang Xia, Jia Tang, Shanhui Liang, Xiaohua Wu

**Affiliations:** ^1^ Department of Gynecologic Oncology, Fudan University Shanghai Cancer Center, Shanghai, PR China; ^2^ Department of Oncology, Shanghai Medical College, Fudan University, Shanghai, PR China

**Keywords:** cervical cancer, para-aortic lymph node, pelvic lymph node, lymphadenectomy, SCCA

## Abstract

Para-aortic lymph node (PALN) dissection is optional and controversial in patients with stage IB1-IIA2 cervical cancer. This retrospective study investigated PALN involvement patterns and evaluated preoperative clinical factors. A total of 723 consecutive FIGO stage IB1-IIA2 cervical cancer patients were included in the study. All patients underwent radical hysterectomy/radical trachelectomy, pelvic lymph node dissection, and PALN dissection. PALN metastasis was found in 101 (14.0%) patients, and the positive PALN rates of stage IB1, IB2, IIA1, and IIA2 were 8.4%, 11.1%, 17.2% and 21.7%, respectively. A multivariate model suggested age > 46 years (OR: 1.67, 95% confidence interval (CI): 1.08-2.58), tumor size > 3.5 cm (OR: 1.79, 95% CI: 1.12-2.87), and FIGO stage IIA (*vs*. IB) (OR: 1.97, 95% CI: 1.25-3.11) all positively correlated with PALN metastasis. When squamous cervical cancer cases were categorically analyzed, a multivariate model indicated age > 46 years (OR: 1.67, 95% CI: 1.00-2.80), FIGO stage IIA (*vs*. IB) (OR: 1.76, 95% CI: 1.02-3.02), and squamous cell carcinoma antigen (SCCA) > 6.5 ng/ml (OR: 5.20, 95% CI: 3.07-8.81) all positively correlated with PALN metastasis. Age, tumor size, and FIGO stage correlated with PALN metastasis in cervical cancer, while age, FIGO stage, and SCCA level were predictive in squamous cell carcinoma.

## INTRODUCTION

The most recent data from GLOBOCAN 2012 showed the global incidence of cervical cancer was about 527,600 with an annual mortality of 265,700 [1]. Each year there are about 98,000 cervical cancer patients newly diagnosed in China [2], thus it is of great importance to optimize individual treatment of cervical cancer.

Lymph node (LN) metastasis is an independent prognostic factor for cervical cancer patients [3, 4]. Typically, the adjacent obturator nodes will be the first site of LN metastasis, then it will spread in a step-wise fashion to the ipsilateral common iliac lymph nodes and para-aortic lymph nodes (PALN). Patients with PALN metastasis have lower survival rates and need extended field radiation. Dissection of enlarged PALN itself could have potential treatment value.

Pelvic lymphadenectomy is recommended by the National Comprehensive Cancer Network (NCCN) to treat cervical cancer [5]. However, the choice of para-aortic lymphadenectomy is usually optional because the incidence of PALN involvement in early stage cancer is low, and it is also difficult to diagnose positive PALN before surgery [6]. One recent meta-analysis concluded that CT and MRI had sensitivities of only 50% and 56%, respectively. PET or PET-CT had much better accuracy with a sensitivity of 82% [7], but patients can have a 15% chance of positive PALN even with a negative PET-CT scan [8]. Moreover, PET-CT could not be widely-used in every patient due to cost efficiency, especially in developing countries.

To identify PALN metastasis predictive factors that can be evaluated before surgery, we conducted this retrospective study in patients with FIGO stage IB1-IIA2 cervical cancer, aiming to discover PALN involvement patterns and optimize the decision process of whether to perform PALN dissection.

## RESULTS

### Patients’ characteristics

A total of 723 consecutive FIGO stage IB1-IIA2 cervical cancer patients who underwent pelvic and para-aortic lymphadenectomy were analyzed in this study. Median age of the population was 45 years (range: 20-76), and the minority of patients (N = 177, 24.5%) were in menopause. Squamous cell carcinoma was the dominating histological type with 639 cases (88.4%). Among all these cases, the median number of PALNs removed was 4 (range: 1 to 41 nodes), 622 (86.0%) patients had negative PALN, and the other 101 (14.0%) had positive PALN. In 81 patients with small tumor size (≤ 2.0 cm), only 4 patients (4.9%) had PALN metastasis. Ovarian metastasis was rare in our study, which only represented 12 cases: 6 with squamous cell cancer (SCC), 5 with adenocarcinoma (AC), and 1 with adenosquamous carcinoma (ACS).

Baseline demographic and clinical characteristics stratified by PALN metastasis are presented in Table 1. Based on best cutoff values of ROC curve, age, tumor size, and SCCA were respectively grouped by > 46 y, > 3.5 cm, and > 6.5ng/ml in this study. Body mass index (BMI) was not obtained for two patients, the tumor size of 11 patients was not measureable, and SCCA was not detected in 105 (16.4%) patients. The two groups did not differ in BMI, eastern cooperative oncology group (ECOG), menopause, childbirth, surgical type, and histological type. However, positive PALN group showed association in age > 46y (*p* = 0.007), advanced FIGO stage (*p* = 0.001), tumor size > 3.5cm (*p* = 0.004), ovarian preservation (*p* < 0.001) and ovarian metastasis (*p* = 0.001).

**Table 1:** Baseline demographic and clinical characteristics of all patients

Feature		PALN negative (%)	PALN positive (%)	*p* value
Total		622(100.0)	101(100.0)	
Age	<=46y	364(58.5)	44(43.6)	0.007
	>46y	258(41.5)	57(56.4)	
BMI	<18.5	50(8.1)	7(6.9)	0.841
	18.5~24.9	448(72.3)	72(71.3)	
	>=25.0	122(19.7)	22(21.8)	
ECOG	0 or 1	571(91.8)	90(89.1)	0.343
	2 or 3	51(8.2)	11(10.9)	
Menopause	No	477(76.7)	69(68.3)	0.080
	Yes	145(23.3)	32(31.7)	
Childbirth	No	33(5.3)	3(3.0)	0.459
	Yes	589(94.7)	98(97.0)	
FIGO stage*	IB1	252(40.5)	23(22.8)	0.001
	IB2	80(12.9)	10(9.9)	
	IIA1	178(28.6)	37(36.6)	
	IIA2	112(18.0)	31(30.7)	
Histological type	SCC	547(87.9)	92(91.1)	0.576
	AC	49(7.9)	5(5.0)	
	ASC	26(4.2)	4(4.0)	
Tumor size	<=3.5cm	271(44.3)	29(29.0)	0.004
	>3.5cm	341(55.7)	71(71.0)	
Ovarian preservation	No	426(68.5)	87(86.1)	<0.001
	Yes	196(31.5)	14(13.9)	
Ovarian metastasis	No	421(98.8)	80(92.0)	0.001
	Yes	5(1.2)	7(8.0)	

Abbreviations: PALN: para-aortic lymph node, BMI: body mass index, ECOG: eastern cooperative oncology group, SCC: squamous carcinoma, AC: adenocarcinoma, ASC: adenosquamous carcinoma

*FIGO stages are defined by the 2009 FIGO (International Federation of Gynaecology and Obstetrics) staging system.

**Figure 1:**
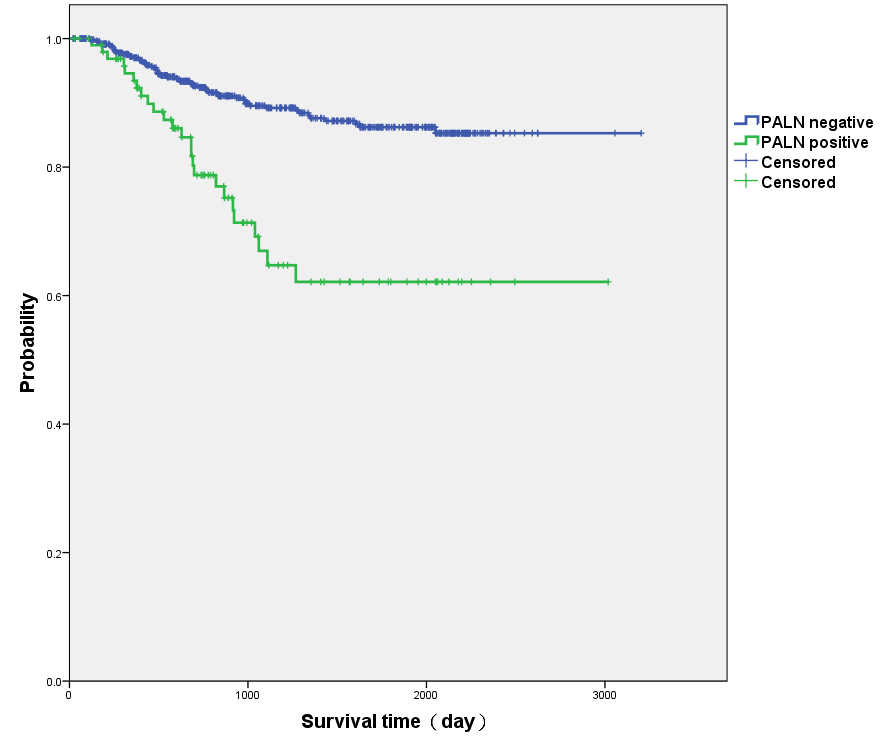
Kaplan-Meier curves of positive para-aortic lymph node (PALN) patients and negative PALN patients Three-year survival rate of negative PALN patients was higher than that of positive PALN patients (89.5 ± 1.5% *vs*. 67.0 ± 6.0%, *p* < 0.001).

The median follow-up time was 34.8 (31.3-38.3) months. Three-year survival rate of negative PALN patients was higher than that of positive PALN patients (89.5±1.5% *vs*. 67.0±6.0%, *p* < 0.001) (Kaplan-Meier curves in Figure 1).

### Lymph node metastasis pattern

The number of cases of IB1, IB2, IIA1, and IIA2 stage cancers were 275, 90, 215, and 143, respectively (Table 2). The corresponding positive PALN rates were 8.4%, 11.1%, 17.2%, and 21.7%, which suggested that patients with more advanced FIGO stage were more likely to have PALN metastasis. PLN involvement rates also increased from 30.9% at the IB1 stage to 59.4% at the IIA2 stage.

**Table 2:** FIGO stage and lymph node metastasis rate

FIGO stage	Number	Positive PLN (%)	Positive PALN (%)
IB1	275	85 (30.9%)	23 (8.4%)
IB2	90	38 (42.2%)	10 (11.1%)
IIA1	215	90 (41.9%)	37 (17.2%)
IIA2	143	85 (59.4%)	31 (21.7%)
Total	723	298 (41.2%)	101 (14.0%)

Abbreviations: FIGO: International Federation of Gynaecology and Obstetrics, PLN: pelvic lymph node, PALN: para-aortic lymph node

Of 298 patients with positive PLN, 98 (32.9%) had PALN metastasis at the same time. Positive PLN was associated with PALN in univariate logistic regression (OR: 68.93, 95% CI: 21.59-220.10, *p* < 0.001). Moreover, in 118 patients with positive common iliac LN, 77 patients (65.3%) were also found positive for PALN metastasis. Twenty-four patients with positive PALN showed no evidence of common iliac LN involvement, and 3 of 101 positive PALN patients (3.0%) had no pelvic nodal disease. There was no uterine body involvement in the three skipped metastasis cases, only one of them had ovarian metastasis.

### Logistic analyses of PALN metastasis

Univariate Logistic analyses suggested that age > 46y, post-menopausal, tumor size > 3.5 cm, and FIGO stage IIA (*vs*. IB) were associated with higher risk of PALN metastasis (*p* < 0.1; Table 3). After a stepwise backward elimination of these 4 potential predictive factors in a multivariate model, 3 covariates remained significant: age > 46y (OR: 1.67, 95% CI: 1.08-2.58; *p* = 0.020), tumor size > 3.5 cm (OR: 1.79, 95% CI: 1.12-2.87; *p* = 0.015) and FIGO stage IIA (*vs*. IB) (OR: 1.97, 95% CI: 1.25-3.11; *p* = 0.003). Positive PALN rate of patients with 0 (*n* = 119), 1 (*n* = 235), 2(*n* = 257) and 3 (*n* = 112) of those risk factors were 5.0%, 9.4%, 17.5% and 25.0%, respectively.

**Table 3:** Logistic regression of preoperative factors associated with para-aortic lymph node metastasis in cervical cancer patients (*N* = 723)

Category		Univariate		Multivariate	
		OR (95% CI)	*P* value	OR (95% CI)	*P* value
Age (y)	>46 *vs*. <=46	1.83 (1.20-2.79)	0.005	1.67 (1.08-2.58)	0.020
Post-menopausal	Yes *vs*. No	1.53 (0.97-2.41)	0.071	0.99 (0.55-1.78)	0.967
Childbirth	Yes *vs*. No	1.83 (0.55-6.08)	0.324		
Tumor size (cm)	>3.5 *vs*. <=3.5	1.95 (1.23-3.08)	0.005	1.79 (1.12-2.87)	0.015
FIGO stage	IIA2+IIA1 *vs*. IB1+IB2	2.36 (1.51-3.68)	<0.001	1.97 (1.25-3.11)	0.003
Histological type	AC *vs*. SCC	0.61 (0.24-1.56)	0.301		
	ASC *vs*. SCC	0.92 (0.31-2.68)	0.871		

Abbreviations: SCC: squamous carcinoma, AC: adenocarcinoma, ASC: adenosquamous carcinoma

### Logistic analyses of PALN metastasis in SCC

When SCC cases were analyzed, SCCA baseline level was taken into consideration (Table 4). The relative predictive factors in univariate logistic analyses were age > 46y, tumor size > 3.5 cm, FIGO stage IIA, and SCCA > 6.5 ng/ml. Further multivariate model indicated 3 covariates: age > 46y (OR: 1.67, 95% CI: 1.00-2.80; *p* = 0.050), FIGO stage IIA (*vs*. IB) (OR: 1.76, 95% CI: 1.02-3.02; *p* = 0.041), and SCCA > 6.5ng/ml (OR: 5.20, 95% CI: 3.07-8.81; *p* < 0.001). The positive PALN rates of patients with 0 (*n* = 153), 1 (*n* = 228), 2 (*n* = 213), and 3 (*n* = 45) of the above risk factors were 5.2%, 9.2%, 22.1%, and 35.6%, respectively (Table 5).

**Table 4:** Logistic regression of preoperative factors associated with para-aortic lymph node metastasis in squamous cell carcinoma patients (*N* = 639)

Category		Univariate		Multivariate	
		OR (95% CI)	*P* value	OR (95% CI)	*P* value
Age (y)	>46 *vs*. <=46	1.68 (1.07-2.62)	0.022	1.67 (1.00-2.80)	0.050
Post-menopausal	Yes *vs*. No	1.26 (0.77-2.07)	0.354		
Childbirth	Yes *vs*. No	2.07 (0.48-8.89)	0.330		
Tumor size (cm)	>3.5 *vs*. <=3.5	2.05 (1.25-3.34)	0.004	1.29 (0.71-2.35)	0.397
FIGO stage	IIA2+IIA1 *vs*. IB1+IB2	2.11 (1.32 -3.36)	0.002	1.76 (1.02-3.02)	0.041
SCCA(ng/ml)	>6.5 *vs*. <=6.5	5.26 (3.13-8.85)	<0.001	5.20 (3.07-8.81)	<0.001

Abbreviations: FIGO: International Federation of Gynecology and Obstetrics, SCCA: squamous cell carcinoma antigen

**Table 5:** Actual rates of para-aortic lymph node metastasis according to the number of risk factors in squamous cell carcinoma patients

Risk factors	Number	Positive PALN (%)
0	153	8 (5.2%)
1	228	21 (9.2%)
2	213	47 (22.1%)
3	45	16 (35.6%)
Total	639	92 (14.4%)

Abbreviations: PALN: para-aortic lymph node

Risk factors: 1. FIGO stage=IIA(vs. IB), 2. age>46y, 3. SCCA>6.5ng/ml.

## DISCUSSION

In this study, we retrospectively inquired into the relationship between clinical factors and PALN metastasis in patients with stage IB1-IIA2 cervical cancer. Results showed that age, tumor size, FIGO stage, and SCCA elevation might indicate higher probability of PALN involvement. To our knowledge, this is the largest population-based analysis of para-aortic lymphadenectomy in cervical cancer.

PALN metastasis is a prognostic factor in cervical cancer [9–11]. A Gynecologic Oncology Group (GOG) study demonstrated that cervical cancer patients had worse 2-year progression-free survival dropping from 64% down to 14% with PALN metastasis [12]. In a prospective multicenter study, 3-year overall survival (OS) of locally advanced cervical cancer patients (FIGO stage IB2-IVA) without PALN metastasis was 89%, while 3-year OS of those with PALN metastasis decreased to 40% [13]. Our survival data also showed that PALN status impacted the 3-year survival rate.

Imaging is widely used in determining lymph node metastasis, but lymph node micrometastasis may not accurately be detected with CT or MRI [14, 15]. Thus we thought it could be more appropriate and reasonable to predict PALN metastasis by combining multiple ways, but not imaging alone. A nomogram model using age, tumor size by MRI, and PET-CT result can predict LN metastasis in early stage cancer [16]. It showed a high negative predictive value of identifying low risk patients whose lymphadenectomy might be unnecessary. However, considering that the majority of their patients were stage IB1 patients (65.9%) whose PALN rate was low, there was no specific demonstration of PALN metastasis rate and PALN prediction accuracy.

Multiple studies proved that incidence of PALN metastasis increased with advancing FIGO stage [17–19]. In a previous study of 621 cervical cancer patients, 5% of stage IB, 16% of Stage II, and 25% of stage III were found PALN metastasis at staging laparotomy [17]. Another study of 208 cases reported the incidence of PALN metastasis in stage IB-IIB was 10-40% [20]. Result in our study showed similar ratio.

In a study by Huang *et al*, no PALN involvement was found in patients with tumor size < 2 cm or negative pelvic lymph node (PLN) metastasis [21]. Our results show 4.9% positive PALN in tumors smaller than 2 cm, which represent 10% of our cases. With our cases with tumor size 3-5 cm, we noticed that 3.5 cm was the most appropriate cutoff point statistically. However, when SCC patients were categorically analyzed, we did not find significant relevance of tumor size with PALN when tumor marker was added into the variables.

SCCA is commonly used to assist in the diagnosis and surveillance of squamous cell cancers, and serum concentration of SCCA is elevated in 28 - 88% of patients with SCC [22, 23]. Compared to patients without lymph node metastasis, SCCA levels before treatment were higher in patients with lymph node metastasis [24]. In a previous study by Bolger *et al*, when pretreatment SCCA level was > 2, > 4, and > 8.6 ng/ml, the positive predictive value for lymph node metastasis was 51.4%, 70.0%, and 100%, with sensitivities of 58.1%, 45.2%, and 22.6%, respectively [25].

In the majority of cases, PALN metastasis is correlated with positive PLN, and skip metastasis is lower than 4%, similar with our results [15, 20, 26]. The incidence of PALN metastasis in our total sample set was 14.0%, but was 32.9% when PLN was involved, and 65.3% when common iliac LN was involved. Tsuruga *et al* obtained similar results, showing that positive PALN rate jumped from 9% to 60% if common iliac LN was positive [9]. One limitation of these studies, including ours, was that most pathology data was collected after the surgery. PLN metastasis might be hard to be accurately diagnosed during surgery, so positive pelvic LN was not designated a preoperative clinical factor in our multivariate logistic analysis.

Lv *et al* utilized intra-operative frozen section of common iliac LN to judge whether PALN dissection was redundant in stage IB1 and IIA1 patients, and showed higher excision accuracy of positive PALN [27]. Theoretically, frozen section of common iliac LN might be efficient to avoid unnecessary PALN dissection, but our study has explored 24 (23.8%) positive PALN cases without common iliac LN metastasis. Other studies have observed 30% absence of common iliac LN metastasis as well [9, 21], suggesting it may be more appropriate to combine intra-operative frozen sections of common iliac LN with other clinical factors for predicting PALN metastasis.

**Figure 2:**
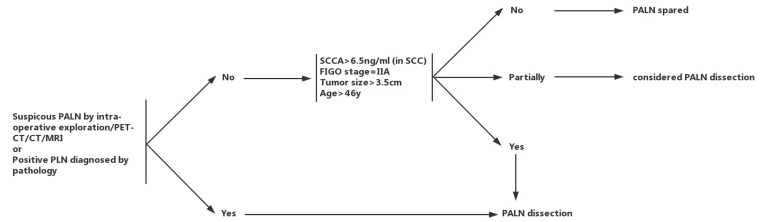
Flow chart of individual para-aortic lymphadenectomy in patients with stage IB1-IIA2 cervical cancer PALN: para-aortic lymph node, PLN: pelvic lymph node (including obturator, external, internal, and common iliac lymph nodes), SCCA: squamous cell carcinoma antigen, SCC: squamous carcinoma.PALN dissection was recommended for patients under any one of the following situations: 1. suspicious PALN by intra-operative exploration/PET-CT/CT/MRI, 2. positive PLN diagnosed by pathology(e.g. frozen section, imprint smear). For the remaining patients, PALN dissection was recommended for those with FIGO stage = IIA, tumor size > 3.5 cm, age > 46y and SCCA > 6.5 ng/ml (in SCC).

According to results of this study and above analysis, we proposed a flow chart to properly identify candidates for para-aortic lymphadenectomy in patients with FIGO stage IB1-IIA2 cervical cancer (Figure 2). PALN dissection was recommended for patients under any one of the following situations: 1. suspicious PALN by intra-operative exploration/PET-CT/CT/MRI, 2. positive PLN diagnosed by pathology(e.g. frozen section, imprint smear). For the remaining patients, PALN dissection was recommended for those with FIGO stage = IIA, tumor size > 3.5 cm, age > 46y and SCCA > 6.5 ng/ml (in SCC). This flow chart might help physicians to distinguish high-risk patients who would benefit most from para-aortic lymphadenectomy during radical hysterectomy.

Our study had several limitations. Firstly, all our patients were recruited from a single center, which could lead to selection bias. Secondly, the present design was a retrospective study. Another possible limitation was the absence of preoperative imaging evaluation, which could also contribute to the choice of para-aortic lymphadenectomy [28]. Taken together, the efficacy of our predictors and decision strategy of PALN dissection could be further evaluated in prospective multi-center trials.

Importantly, we found that age > 46y, tumor size > 3.5 cm, and FIGO stage IIA (*vs*. IB) were positively correlated with PALN metastasis in cervical cancer. In squamous cell carcinoma, age > 46y, FIGO stage IIA (*vs*. IB), and SCCA level > 6.5 ng/ml were identified as predictive factors for positive PALN. These factors can be detected easily before surgery, help to make optimum clinical decisions during radical hysterectomy.

## MATERIALS AND METHODS

### Patients

We conducted a retrospective study of first diagnosed cervical cancer patients who underwent PALN dissection in Fudan University Shanghai Cancer Center from January 2006 to February 2014. To eliminate the interference of confounding factors, we defined the inclusion criteria as: cervical cancer FIGO stage Ib1-IIa2, no neoadjuvant chemotherapy or radiotherapy, no second primary tumor when diagnosed, designated histological type (SCC, AC, and ACS), and complete radical surgery. After obtaining approval from our institutional review board, data was reviewed and a total of 723 patients fulfilling the above criteria were enrolled in this study. All patients signed informed written consent to therapeutic interventions (including surgery, chemotherapy, and radiotherapy if necessary).

Patients’ medical records were collected, such as age, body mass index, ECOG performance status, menstrual status, baseline SCCA level, FIGO stage, tumor size, histological report, surgical procedure, ovarian metastasis, and pelvic and para-aortic lymph node status. Tumor size was measured during surgery; squamous cell carcinoma antigen (SCCA) measurement was performed using an ARCHITECT i 2000SR (Abbott, Illinois, U.S.A). The normal range is 0-1.5 ng/ml.

### Treatment

Gynecologic oncologists from a single center (Fudan University Shanghai Cancer Center) performed all of the radical surgeries to maintain consistency. Type III hysterectomy of Piver-Rutledge classifications was performed by laparotomy in 714 cases. The remaining 9 patients who were willing and eligible to maintain future fertility underwent abdominal radical trachelectomy as previously demonstrated [29]. All the 723 patients had systematic pelvic and para-aortic lymphadenectomy during the radical surgery procedure. Pelvic lymph nodes included the most common lymphatic pathways, such as the obturator, external, internal, and common iliac lymph nodes. PALN dissection was usually conducted along the flow of the abdominal lymphatics until reaching the inferior mesenteric artery (IMA). When infra-IMA node metastasis was suspected by preoperative evaluation or intraoperative exploration, PALNs were further resected until reaching the left renal vein.

Adjuvant treatment was used after the radical surgery when patients had high or intermediate risk prognostic factors, including positive LN, positive margin, positive parametria, large tumor size, deep stromal invasion, and lymphovascular space invasion. Pelvic radiation at 40-50 Gy was offered with or without concurrent cisplatin-based chemotherapy individually. When PALN were involved, extended-field radiation including PLN and PALN was recommended. Platinum-based chemotherapy alone was occasionally used in some specific patients who rejected radiotherapy.

### Statistical methods

Survival curves were done by the Kaplan-Meier method and analyzed by the log-rank test. The χ2 test was used to assess differences in the distributions of demographic characteristics between positive PALN cases and negative PALN cases. The association between relevant factors and PALN metastasis was assessed by odds ratio (OR) and 95% confidence interval (CI). OR values were calculated by both univariate and multivariate logistic regression models, which was applied using stepwise backward elimination until only significant (*p* < 0.05) variables remained in multivariate survival analysis. All statistical analyses were performed using SPSS version 13.0 statistical software (SPSS Inc., Chicago, IL). A *p* value of < 0.05 was considered statistically significant.
